# Supplementary material for: Conserved wing shape variation across biological scales unveils dialectical relationships between micro- and macroevolution
Source: Commun Biol. 2025 Jul 7;8:990. doi: 10.1038/s42003-025-08376-2 (PMC12234666; doi:10.1038/s42003-025-08376-2)
Supplement: Supplementary file 1 — Supplymentary Information [file 42003_2025_8376_MOESM1_ESM.pdf]

Supplementary information

Conserved wing shape variation across biological scales unveils dialectical relationships between micro- and macroevolution

Keita Saito[1], Masahito Tsuboi[2*] and Yuma Takahashi[3†]

[1]Graduate School of Science and Engineering, Chiba University, Chiba, 263-8522, Japan

[2]Department of Biology, Lund University, Lund, Sweden

[3]Graduate School of Science, Chiba University, Chiba, 263-8522, Japan

*Corresponding Author

Address: Sölvegatan 37, 22362 Lund, Sweden

Phone: +46733868631

Email: masa.tsuboi@gmail.com

†Corresponding Author

Address: 1-33, Yayoi, Inage, Chiba, Chiba, 263-8522 Japan

Phone: +81-43-290-3965

Email: takahashi.yum@gmail.com

Masahito Tsuboi and Yuma Takahashi contributed equally.

Contents

Supplementary Note 1

Supplementary Fig. 1

Supplementary Fig. 2

Supplementary Fig. 3

Supplementary Table 1

Supplementary Table 2

Supplementary Table 3

Supplementary Table 4

Supplementary Table 5

References

Supplementary Note 1

Measuring **M** in *D. simulans* would provide more accurate results. However, estimating **M** requires Mutation Accumulation (MA) experiments, which are time-consuming in *Drosophila* due to their relatively long generation time compared to organisms such as *Escherichia coli*, which are more commonly used for MA experiments. Therefore, in this study, we used **M** estimated from *D. melanogaster*, a species closely related to *D. simulans*. In addition, **F** could not be estimated from the Houle group's data because they did not measure both left and right wings, which is essential for calculating fluctuating asymmetry. As a result, **F** and **M** could not be compared within the same species.

$\mathbf{M}_{hom}$ represents the mutational co/variance matrix estimated under homozygous conditions, while $\mathbf{M}_{het}$ is estimated under heterozygous conditions[1]. The former captures the full phenotypic effects of spontaneous mutations without genetic masking, thus providing a direct and upper-bound measure of mutational input. In contrast, the latter reflects the phenotypic effects of new mutations expressed in heterozygous individuals, which more closely approximates how mutations contribute to phenotypic variation and are acted upon by natural selection in outbred populations. In this study, we primarily focus on $\mathbf{M}_{hom}$ because it more directly reflects the variability introduced by spontaneous mutations and thus provides a clearer index of mutational input. However, we also include $\mathbf{M}_{het}$ in our analysis to enable a more comprehensive understanding of mutational variance in both experimental and natural contexts. Given that natural populations are typically actually expressed and subject to selection in nature. furthermore, our inclusion of both matrices follows the approach adopted by Houle et al.[2].

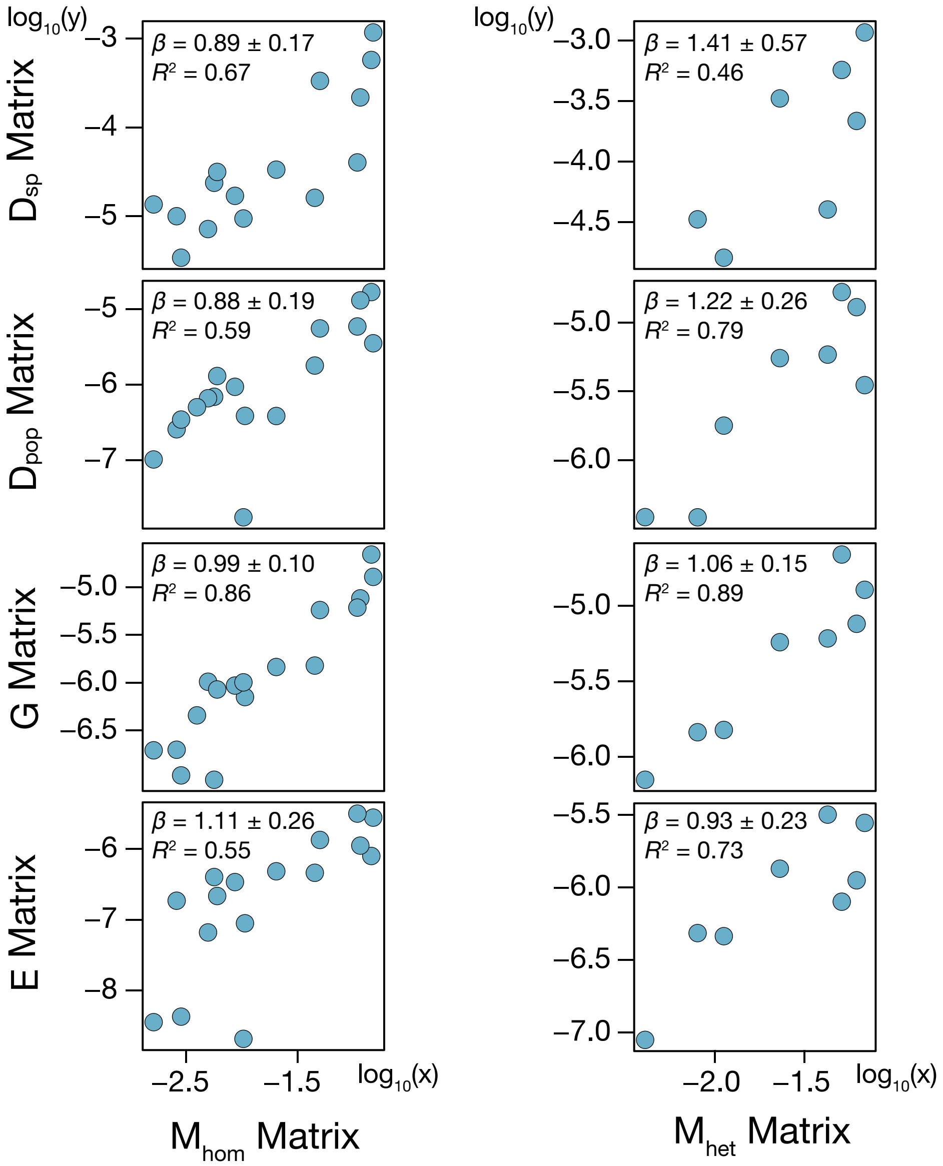

Supplementary Fig. 1. Relationships between mutational variations in *Drosophila melanogaster* ($\mathbf{M_{hom}}$ and $\mathbf{M_{het}}$) and our estimated matrix (E, G, $\mathbf{D_{pop}}$, and $\mathbf{D_{sp}}$). Points represent $\log_{10}$ (variance in each matrix) along the eigenvectors of **G** in *Drosophila melanogaster*. Key gives log–log regression result, β ± S.E. and R^2. Only upper 17 dimensions of

$\mathbf{M}_{hom}$ and eight dimensions of $\mathbf{M}_{het}$ were used. We did not compare both **M** with **R** because these relationships were already revealed in Houle et al. 2018.

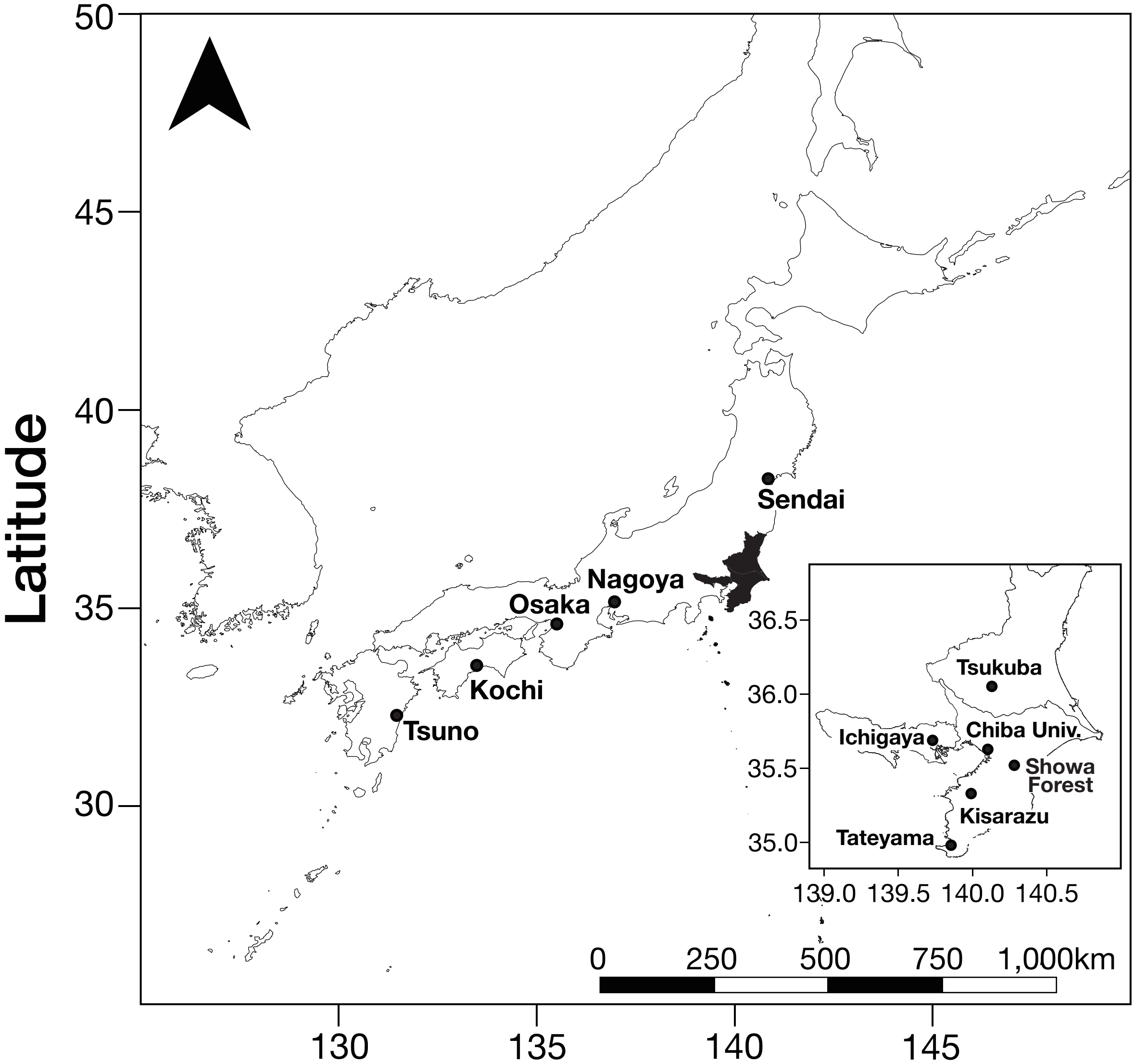

Supplementary Fig. 2. The location of sampling points.

The prefectures shaded in black on the map are enlarged in the inset figure. The map created using public domain data from Natural Earth and vector data from the National Land Numerical

Information download service (Japan Ministry of Land, Infrastructure, Transport and Tourism, MLIT). The original data were modified for visualization.

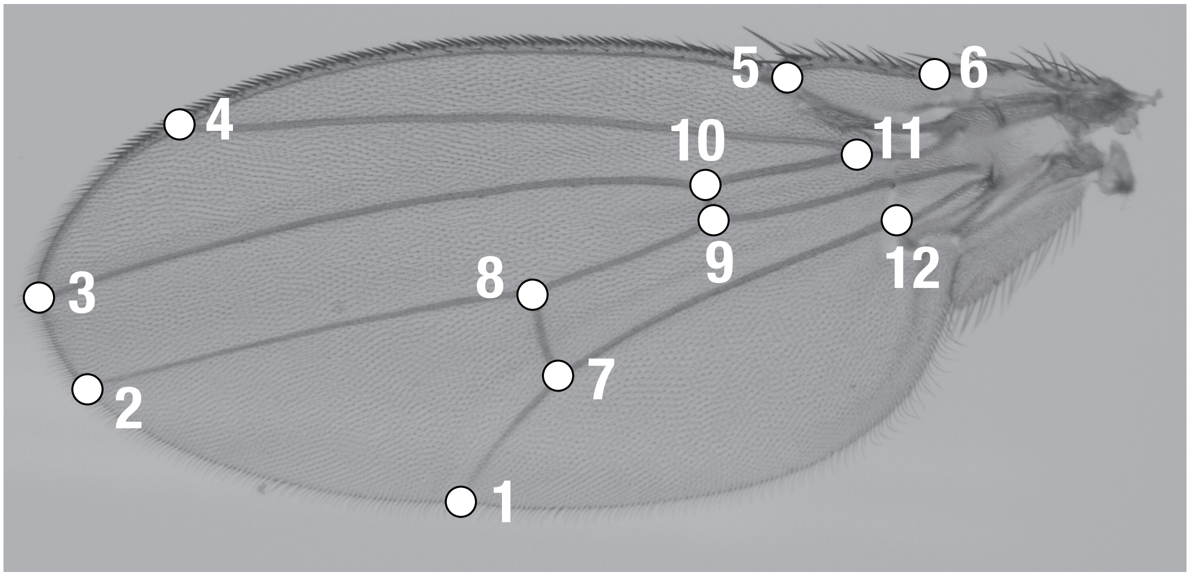

Supplementary Fig. 3. Position of 12 two-dimensional landmarks utilized in this study.

Supplementary Table 1. Pearson's correlation coefficients of pair-wise relationships between $\log_{10}$ variances in all six matrices estimated on the basis of MCMCglmm.

Matrix	1	2	3	4	5	6
1. **R**	–					
2. $\mathbf{D}_{sp}$	0.87^{***}	–				
3. $\mathbf{D}_{pop}$	0.78^{***}	0.77^{***}	–			
4. **G**	0.86^{***}	0.83^{***}	0.76^{***}	–		
5. **E**	0.70^{***}	0.67^{**}	0.80^{***}	0.66^{**}	–	
6. **F**	0.93^{***}	0.87^{***}	0.84^{***}	0.81^{***}	0.79^{***}	–

** $P < 0.01$

*** $P < 0.001$

Supplementary Table 2. Sampling points where we chaptered *D. simulans* and the number of isofemale lines used in this study.

Sampling city	Isofemale lines	Latitude	Longitude
Chiba (Chiba University)	33	35° 62′ 79″ N	140° 10′ 31″ E
Chiba (Showa Forest)	6	35° 52′ 03″ N	140° 28′ 19″ E
Ichigaya	1	35° 68′ 85″ N	139° 73′ 09″ E
Kisarazu	4	35° 32′ 97″ N	139° 99′ 08″ E
Kochi	5	33° 54′ 91″ N	133° 48′ 68″ E
Nagoya	3	35° 15′ 40″ N	136° 96′ 83″ E
Osaka	7	34° 59′ 58″ N	135° 50′ 97″ E
Sendai	5	38° 26′ 10″ N	140° 84′ 95″ E
Tateyama	2	34° 98′ 15″ N	139° 85′ 56″ E
Tsukuba	4	36° 05′ 22″ N	140° 13′ 06″ E
Tsuno	1	32° 28′ 80″ N	131° 46′ 51″ E

Supplementary Table 3. The 8 environment combinations for estimating **E**.

	Nutrients condition	Light-dark cycle	Temperature
Standard	High	12 h light/12 h dark	25°C
Environment 1	Intermediate	12 h light/12 h dark	23°C
Environment 2	High	12 h light/12 h dark	23°C
Environment 3	Low	12 h light/12 h dark	23°C
Environment 4	Intermediate	10 h light/14 h dark	23°C
Environment 5	Intermediate	14 h light/10 h dark	23°C
Environment 6	Intermediate	12 h light/12 h dark	20°C
Environment 7	Intermediate	12 h light/12 h dark	26°C

Supplementary Table 4. Species name and the number of wing photos.

Species	N*	N†
Drosophila acutissima	20	
D. annulipes	31	
D. bizonata	31	
D. busckii	35	
D. coracina	5	
D. curviceps	20	
D. hydei	11	
D. immigrans	41	
D. lutescens	14	1233
D. rufa	41	
D. sexvittata	41	
D. simulans		3982
D. sternopleuralis	42	
D. suzukii	22	

*The number of photos from DrosoWing Project.

†The number of photos from our own experiments.

Supplementary Table 5. The estimate of directional asymmetry.

trait	post.mean	l-95% CI	u-95% CI
traitx1:sideR	0.0003793	0.0001893	0.0005748
traity1:sideR	0.00005429	-0.000237	0.0003266
traitx2:sideR	-4.402E-05	-0.0002717	0.0001972
traity2:sideR	-0.0004754	-0.0006093	-0.0003383
traitx3:sideR	0.00009469	-0.000185	0.0003652
traity3:sideR	0.00028	0.0001203	0.000449
traitx4:sideR	0.0003155	-0.0000436	0.0006064
traity4:sideR	0.0004039	0.0002326	0.0005944
traitx5:sideR	-0.0002957	-0.000466	-0.0001362
traity5:sideR	0.0004851	0.0002727	0.000704
traitx6:sideR	-0.000173	-0.0003447	4.827E-06
traity6:sideR	0.00007076	-0.0001411	0.0002874
traitx7:sideR	-5.875E-05	-0.0003682	0.0002545
traity7:sideR	-0.001138	-0.001441	-0.0007987
traitx8:sideR	-0.0004169	-0.0008274	-8.594E-05
traity8:sideR	0.0006223	0.0004694	0.0007823
traitx9:sideR	0.0001203	-4.507E-05	0.0002759
traity9:sideR	0.0005425	0.0004631	0.0006481
traitx10:sideR	0.00003854	-0.0001542	0.0002359
traity10:sideR	-0.0004269	-0.0005074	-0.0003388
traitx11:sideR	0.0002962	0.0001665	0.0004347
traity11:sideR	0.00007966	-2.297E-05	0.0001982
traitx12:sideR	-0.0002553	-0.0003717	-0.0001704

traity12:sideR	-0.0004988	-0.0006166	-0.0004048
traitx1	-0.1347	-0.135	-0.1344
traity1	-0.2	-0.2003	-0.1998
traitx2	-0.4414	-0.4416	-0.4413
traity2	-0.02596	-0.02612	-0.02582
traitx3	-0.4656	-0.4658	-0.4654
traity3	0.05452	0.05435	0.0547
traitx4	-0.2987	-0.299	-0.2983
traity4	0.1833	0.183	0.1836
traitx5	0.2249	0.2248	0.2251
traity5	0.09948	0.0993	0.09967
traitx6	0.3555	0.3553	0.3556
traity6	0.06687	0.06669	0.06703
traitx7	-0.03186	-0.03216	-0.03157
traity7	-0.1099	-0.1102	-0.1097
traitx8	-0.03882	-0.03915	-0.03851
traity8	-0.03994	-0.04007	-0.03981
traitx9	0.1378	0.1376	0.138
traity9	-0.01183	-0.01189	-0.01175
traitx10	0.1363	0.136	0.1365
traity10	0.01563	0.01555	0.0157
traitx11	0.2616	0.2615	0.2617
traity11	0.01798	0.01789	0.01805
traitx12	0.295	0.2949	0.2952
traity12	-0.05005	-0.05013	-0.04996

References

1. Houle, D. & Fierst, J. Properties of spontaneous mutational variance and covariance for wing size and shape in *Drosophila melanogaster*. *Evolution* **67**, 1116–1130 (2013).
2. Houle, D., Bolstad, G. H., van der Linde, K. & Hansen, T. F. Mutation predicts 40 million years of fly wing evolution. *Nature* **548**, 447–450 (2017).
