# Supplementary material for: Conserved wing shape variation across biological scales unveils dialectical relationships between micro- and macroevolution
Source: Commun Biol. 2025 Jul 7;8:990. doi: 10.1038/s42003-025-08376-2 (PMC12234666; doi:10.1038/s42003-025-08376-2)
Supplement: Supplementary file 2 — Description of Additional Supplementary Files [file 42003_2025_8376_MOESM2_ESM.docx]

Description of Additional Supplementary Files

**File name:** Supplementary Data

**Description:** Source data used to generate the graphs presented in the main figures.
