# Supplementary material for: Conserved wing shape variation across biological scales unveils dialectical relationships between micro- and macroevolution
Source: Commun Biol. 2025 Jul 7;8:990. doi: 10.1038/s42003-025-08376-2 (PMC12234666; doi:10.1038/s42003-025-08376-2)
Supplement: Supplementary file 4 — Reporting Summary [file 42003_2025_8376_MOESM4_ESM.pdf]

nature portfolio

Corresponding author(s): Masahito Tsuboi, Yuma Takahashi

Last updated by author(s): Jun 9, 2025

Reporting Summary

Nature Portfolio wishes to improve the reproducibility of the work that we publish. This form provides structure for consistency and transparency in reporting. For further information on Nature Portfolio policies, see our Editorial Policies and the Editorial Policy Checklist.

Statistics

For all statistical analyses, confirm that the following items are present in the figure legend, table legend, main text, or Methods section.

n/a	Confirmed	
☐	☒	The exact sample size (*n*) for each experimental group/condition, given as a discrete number and unit of measurement
☐	☒	A statement on whether measurements were taken from distinct samples or whether the same sample was measured repeatedly
☐	☒	The statistical test(s) used AND whether they are one- or two-sided<br>*Only common tests should be described solely by name; describe more complex techniques in the Methods section.*
☐	☒	A description of all covariates tested
☒	☐	A description of any assumptions or corrections, such as tests of normality and adjustment for multiple comparisons
☐	☒	A full description of the statistical parameters including central tendency (e.g. means) or other basic estimates (e.g. regression coefficient) AND variation (e.g. standard deviation) or associated estimates of uncertainty (e.g. confidence intervals)
☒	☐	For null hypothesis testing, the test statistic (e.g. *F*, *t*, *r*) with confidence intervals, effect sizes, degrees of freedom and *P* value noted<br>*Give P values as exact values whenever suitable.*
☐	☒	For Bayesian analysis, information on the choice of priors and Markov chain Monte Carlo settings
☒	☐	For hierarchical and complex designs, identification of the appropriate level for tests and full reporting of outcomes
☒	☐	Estimates of effect sizes (e.g. Cohen's *d*, Pearson's *r*), indicating how they were calculated

Our web collection on statistics for biologists contains articles on many of the points above.

Software and code

Policy information about availability of computer code

Data collection	ml-morph (Porto and Voje, 2020)
Data analysis	R (version4. 1. 2)

For manuscripts utilizing custom algorithms or software that are central to the research but not yet described in published literature, software must be made available to editors and reviewers. We strongly encourage code deposition in a community repository (e.g. GitHub). See the Nature Portfolio guidelines for submitting code & software for further information.

Data

Policy information about availability of data

All manuscripts must include a data availability statement. This statement should provide the following information, where applicable:

- Accession codes, unique identifiers, or web links for publicly available datasets
- A description of any restrictions on data availability
- For clinical datasets or third party data, please ensure that the statement adheres to our policy

Figshare, 10.6084/m9.figshare.26861356

Research involving human participants, their data, or biological material

Policy information about studies with human participants or human data. See also policy information about sex, gender (identity/presentation), and sexual orientation and race, ethnicity and racism.

Reporting on sex and gender	n/a
Reporting on race, ethnicity, or other socially relevant groupings	n/a
Population characteristics	n/a
Recruitment	n/a
Ethics oversight	n/a

Note that full information on the approval of the study protocol must also be provided in the manuscript.

Field-specific reporting

Please select the one below that is the best fit for your research. If you are not sure, read the appropriate sections before making your selection.

☐ Life sciences ☐ Behavioural & social sciences ☒ Ecological, evolutionary & environmental sciences

For a reference copy of the document with all sections, see nature.com/documents/nr-reporting-summary-flat.pdf

Ecological, evolutionary & environmental sciences study design

All studies must disclose on these points even when the disclosure is negative.

Study description	In this study, we collected and analyzed quantitative traits data (fruit flies' wing) and examined the correlations between different biological levels (species divergence, population divergence within a species, genetic variation within a species, phenotypic plasticity, mutational variance, developmental noise).
Research sample	Fruit Flies
Sampling strategy	Sampling of Drosophila (especially, Drosophila simulans) was conducted at multiple locations across Japan. Female adults were collected and inbred for multiple generations under laboratory conditions to establish inbred lines (iso-female lines). These iso-female lines were then maintained in the laboratory environment. While the minimum number of iso-female lines per location was not determined, the minimum number of individuals per an iso-female line was set to be at least 3 individuals
Data collection	Data collection was carried out by Keita Saito. Wings from multiple individuals of the established iso-female lines were dry-mounted to create wing specimens. Afterward, photographs of the wings were taken while cross-referencing the specimen ID and other information (such as line number, rearing conditions, wing sides, sampling location, etc.). The same procedure was followed when photographing data (photos) from previously published papers.
Timing and spatial scale	There is no consistency regarding the timing or season of the sampling. Since the individuals were reared for a certain number of generations in the laboratory environment, I interpret that there are no issues in terms of environmental effects being eliminated. Additionally, there is no consistency in the timing of specimen preparation, as it has been carried out throughout the year, starting from the lines for which iso-female line creation was completed. Data collection itself was conducted year-round, from the winter of 2020 to the spring of 2023.
Data exclusions	If there is significant damage that makes it impossible to identify the landmarks of wing when taking photographs, the data from that specimen has not been used.
Reproducibility	Photographs of all specimens were taken twice, and measurement errors during specimen photography and wing quantification were quantified.
Randomization	Group allocation was determined based on factors such as which population the specimens were from, the rearing conditions, and the origin of the iso-female line. When quantifying the target variables, other variables were included as random effects for estimation
Blinding	n/a

Did the study involve field work? ☒ Yes ☐ No

Field work, collection and transport

Field conditions	A typical climate of a Japanese city.
Location	This is summarized in Supplementary Fig.2 and Supplementary Table 2.
Access & import/export	All sampling was conducted in compliance with Japanese law.
Disturbance	n/a

Reporting for specific materials, systems and methods

We require information from authors about some types of materials, experimental systems and methods used in many studies. Here, indicate whether each material, system or method listed is relevant to your study. If you are not sure if a list item applies to your research, read the appropriate section before selecting a response.

Materials & experimental systems

n/a	Involved in the study	
☒	☐	Antibodies
☒	☐	Eukaryotic cell lines
☒	☐	Palaeontology and archaeology
☐	☒	Animals and other organisms
☒	☐	Clinical data
☒	☐	Dual use research of concern
☒	☐	Plants

Methods

n/a	Involved in the study	
☒	☐	ChIP-seq
☒	☐	Flow cytometry
☒	☐	MRI-based neuroimaging

Animals and other research organisms

Policy information about studies involving animals; ARRIVE guidelines recommended for reporting animal research, and Sex and Gender in Research

Laboratory animals	Do not include laboratory animals.
Wild animals	The main target species is Drosophila simulans. Sampling was conducted either through sweeping or by traps with bananas.
Reporting on sex	No distinction was made based on sex during sampling. For the experiment, only female adults were used to minimize the effects derived from sexual dimorphism.
Field-collected samples	The rearing conditions are described in the Methods section. After the experiment, the samples were frozen to halt biological activity and then incinerated for disposal.
Ethics oversight	No ethical approval or guidance was required.

Note that full information on the approval of the study protocol must also be provided in the manuscript.

Plants

Seed stocks	*Report on the source of all seed stocks or other plant material used. If applicable, state the seed stock centre and catalogue number. If plant specimens were collected from the field, describe the collection location, date and sampling procedures.*
Novel plant genotypes	*Describe the methods by which all novel plant genotypes were produced. This includes those generated by transgenic approaches, gene editing, chemical/radiation-based mutagenesis and hybridization. For transgenic lines, describe the transformation method, the number of independent lines analyzed and the generation upon which experiments were performed. For gene-edited lines, describe the editor used, the endogenous sequence targeted for editing, the targeting guide RNA sequence (if applicable) and how the editor was applied.*
Authentication	*Describe any authentication procedures for each seed stock used or novel genotype generated. Describe any experiments used to assess the effect of a mutation and, where applicable, how potential secondary effects (e.g. second site T-DNA insertions, mosiacism, off-target gene editing) were examined.*
